# A high performance profile-biomarker diagnosis for mass spectral profiles

**DOI:** 10.1186/1752-0509-5-S2-S5

**Published:** 2011-12-14

**Authors:** Henry Han

**Affiliations:** 1 Department of Mathematics and Bioinformatics, Eastern Michigan University, Ypsilanti MI, 48197, USA; 2 The Laboratory for High Performance Computing in Bioinformatics, Eastern Michigan University, Ypsilanti, MI 48197, USA

## Abstract

**Background:**

Although mass spectrometry based proteomics demonstrates an exciting promise in complex diseases diagnosis, it remains an important research field rather than an applicable clinical routine for its diagnostic accuracy and data reproducibility. Relatively less investigation has been done yet in attaining high-performance proteomic pattern classification compared with the amount of endeavours in enhancing data reproducibility.

**Methods:**

In this study, we present a novel machine learning approach to achieve a clinical level disease diagnosis for mass spectral data. We propose multi-resolution independent component analysis, a novel feature selection algorithm to tackle the large dimensionality of mass spectra, by following our local and global feature selection framework. We also develop high-performance classifiers by embedding multi-resolution independent component analysis in linear discriminant analysis and support vector machines.

**Results:**

Our multi-resolution independent component based support vector machines not only achieve clinical level classification accuracy, but also overcome the weakness in traditional peak-selection based biomarker discovery. In addition to rigorous theoretical analysis, we demonstrate our method’s superiority by comparing it with nine state-of-the-art classification and regression algorithms on six heterogeneous mass spectral profiles.

**Conclusions:**

Our work not only suggests an alternative direction from machine learning to accelerate mass spectral proteomic technologies into a clinical routine by treating an input profile as a ‘profile-biomarker’, but also has positive impacts on large scale ‘omics' data mining. Related source codes and data sets can be found at: https://sites.google.com/site/heyaumbioinformatics/home/proteomics

## Background

With recent surges in proteomics, mass spectral proteomic pattern diagnostics has become a highly promising way of diagnosing, predicting, and monitoring cancers or other advanced diseases for its cost-effectiveness and efficiency [1]. Recent studies not only demonstrate that proteomic profiling can detect the anonymous protein peaks differently expressed between cancer patients and healthy subjects, but also show the absence or presence of disease can be discovered through proteomic pattern classification. However, this novel technology remains an important research field rather than a clinical routine because of the unresolved problems in data reproducibility and classification. The data reproducibility issue refers to that no two independent studies have been found to produce same proteomic patterns. On the other hand, the data classification issue refers to that the classification accuracy obtained from mass spectral data is inadequate to attain a clinical level (e.g., 99.5%) in most studies. Although impressive sensitivities and specificities were reported in some case studies, their classification methods have no guarantee to extend to other mass spectral data to maintain a same level performance.

Many methods and protocols are proposed and being developed to enhance mass spectral data reproducibility from biological and technological aspects. They include employing peptide profiling to replace proteomics profiling to get extremely high resolution data, improving experimental designs to avoid mingles between biological and technological variables, and developing more robust preprocessing algorithms [2-5]. However, mass spectral data reproducibility enhancement seems to be facing a built-in challenge from the technology itself [6], i.e., almost any small, even tiny changes in the part of proteome will be amplified to rather large even huge differences in mass spectra, no matter whether the sources of the changes are from biological factors or experimental conditions. The sensitive signal amplification mechanism somewhat limits the potential of these reproducibility enhancement techniques and presents difficulties in achieving reproducible and consistent diagnosis.

On the other hand, rather fewer studies have been invested in improving mass spectral proteomic pattern classification than those of enhancing data reproducibility. To attain high disease diagnostic accuracy, many studies focus on identifying biomarkers from mass spectral profiles, which are generally a small set of protein expression peaks at selected m/z (mass/charge) ratios, through different machine learning approaches (e.g., peak selection), [7,8]. These studies are definitely important and interesting. However, they bear the following limitations. (1) The biomarker selection processing is generally individual data oriented case study. There is no guarantee to generalize it to other profiles. (2) The biomarkers obtained from these studies by nature are not reproducible because of the irreproducibility of their source data. In other words, the identified mass spectral biomarkers may lose their reusability and predictability, even if they can achieve exceptional sensitivity and specificity in classification. It is highly likely that another totally different set of biomarkers would be identified if the same type of mass spectra were generated from another set of cancer patients and healthy individuals under the same experimental conditions. (3) The sensitivity and specificity levels from the biomarkers’ classification are still inadequate to qualify this young technology as a robust clinical routine.

How could we accelerate mass spectral proteomics to become a clinical routine in complex disease diagnosis while the studies on data reproducibility enhancement are still underway? We address this challenge from a machine-learning viewpoint by developing a high-performance mass spectral pattern recognition algorithm in this study. Although data reproducibility plays a very important role in mass spectral proteomics, the essential factor to determine whether this exciting technology can fully explore its potential, to a large degree, may rely on the levels of sensitivity and specificity from mass spectral pattern classification.

If there exists a novel pattern recognition algorithm able to attain a 99.5% level accuracy in mass spectra classification for an input proteomic profile, then the profile can be viewed as a *profile biomarker* in disease diagnosis. This is because the high-accuracy diagnostic results would be reproducible for all input profiles by taking advantage of the novel classification technique. Under such a situation, the data reproducibility probably may not be a major concern to prevent reproducible biomarker discovery because the profile biomarker is able to “reproduce itself” by attaining clinical level diagnosis.

The high or even huge dimensionality of mass spectral data presents a challenge for high-performance proteomic pattern classification, especially for most traditional classification algorithms that were developed under the assumption that input data with a small or medium dimensionality. A mass spectral profile can be represented as a *p* × *n* matrix after preprocessing, where a row represents the ion-intensities of a set of observations (samples) at a mass charge ratio (m/z), which is similar to a gene in microarray data, and a column represents the ion-intensities of a single sample across a set of m/z ratios. Unlike traditional data (e.g., financial data), the number of variables in a mass spectral profile is much greater than the number of observations, i.e., *n*>>*p*. In addition, only a small portion of testing points (m/z ratios) among the thousands of them have meaningful contribution to data variations or demonstrate biological relevance in disease detection. Furthermore, mass spectral data by nature are not-noise free due to the non-linearity in proteomic profiling. Preprocessing techniques are unable to remove some built-in systematic noise completely. The information redundancy, noise, and high-data dimensionalities in mass spectral data not only make some traditional classification methods (e.g., Fisher discriminant analysis) lose discriminative power, but also present an urgent challenge in computational proteomics.

### Local features and global features

Many feature selection methods are employed to decrease dimensionalities, remove noise, and extract meaningful features before mass spectra classification. These methods can be categorized as input-space feature selection and subspace feature selection. The input-space feature selection reduces the dimensionality of data by selecting a subset of features to conduct a hypothesis testing or create a model under some selection criteria in the same space as input data (e.g., t-test). On the other hand, the subspace feature selection, also called transform-based feature selection, reduces data dimensionality by transforming data into a low-dimensional subspace induced by a linear or nonlinear transformation. The subspace feature selection methods are probably the most used data reduction techniques in proteomics for their popularity and efficiency. They include principal component analysis (PCA) [9], independent component analysis (ICA) [10,11], nonnegative matrix factorization (NMF) [12], and their different extensions [13,14]. We mainly focus on the subspace feature selection methods in this study.

These algorithms, however, are generally good at selecting global features rather than local features. The global and local features consist of high frequency and low frequency features (signals) respectively. For example, a testing point (an m/z ratio) with several exceptionally high peaks on cancer samples, which are seldom found at most testing points, can be viewed as a local feature. On the other hand, a testing point whose expression value plot curve is similar to those of other testing points is a global feature. As different frequency signals capturing different data behaviour, the global and local features interpret the global and local behaviour of data, and contribute to the global and local characteristics of data respectively. Since there is no robust screening mechanism available to distinguish the two types of features in most subspace feature selection methods, the global features may demonstrate ‘obvious’ advantages over the local features in the feature selection. That is, the low frequency signals have less likelihood to contribute to the inferred low-dimensional data, which usually are the linear combinations of all input variables, than the high frequency signals. For example, the positive and negative weights in the linear combination to calculate each principal component in PCA are likely to partially cancel each other. However, it causes that the weights representing contributions from local features are more likely to be cancelled out because of their frequencies. As such, unlike the global features, the local features are hard to extract for most subspace feature-selection algorithms. Finally, the low dimensional data inferred from the transform-based feature selection may miss some local data-characteristics described by the local features. In other words, the global features dominate the feature selection and these algorithms demonstrate a *global feature selection mechanism.*

Although difficult to extract out, the local features are probably the key to attaining a high-performance mass spectral pattern classification for its subtle data behaviour capturing, especially because many mass spectral samples share very similar global characteristics but different local characteristics. For example, it’s easy to distinguish a 10-years old, five-feet girl Jean between a 25-year old six-feet male Mike, because they have different global features. However, it is not easy to distinguish Mike with his twin brother Peter because they share almost same global characteristics: height, weight, hair color, *etc*. Nevertheless, some careful people can still detect them because Peter has a mole near his mouth but Mike does not, i.e., the mole here works as the local feature to facilitate such detection. For another example, some benign tumor samples may display very similar global characteristics but quite different local characteristics with malignant tumor samples. To attain a high-accuracy diagnosis, it is must to capture the local data characteristics to distinguish these samples sharing the similar global characteristics from each other. It may be particularly important in mass spectral proteomics because some sub-type samples may demonstrate very similar ‘global patterns’ under the same profiling technology.

### Reasons for the global feature selection mechanism

A major reason for the global feature selection mechanism displayed in these algorithms is that there is no screening technique available to separate two types of features in feature selection. In other words, PCA, ICA, NMF, and their variants all belong to a single-resolution feature selection method, where all features are indistinguishably analyzed in a single-resolution despite the nature of their frequencies. Such an indistinguishable treatment causes the most-often data entries to have a high likelihood to dominate feature selection and the less-often data entries may lose opportunities. In other words, the global features are more likely to be selected than the local features and prevents effective local data-characteristics capturing. As such, the low dimensional data inferred from these methods (e.g., the projection data onto the three principal components in PCA) may probably only demonstrate the global data characteristics. Obviously, the mass spectral samples with similar global characteristics but different local characteristics will not be recognized in the following classification. Moreover, the global feature selection mechanism may bring redundant global features in the following classification because almost only the features that interpreting global characteristics are involved in training the corresponding learning machine (e.g., SVM). The redundant global features will unavoidably decrease the generalization of the learning machine and increase the risk of misclassifications or over-fitting. Finally, the learning machines integrated with the global feature selection algorithms will display instabilities in classifications, i.e., they may perform well on some data but fail badly on the others due to different contributions of the global features to the classification.

To avoid the global feature selection mechanism, it is desirable to distinguish features (e.g., sort) according to their frequencies by building some screening techniques to separate two types of features in the feature selection. In this study, we conduct multi-resolution data analysis via a discrete wavelet transform (DWT) [15] to separate features according to their frequencies. The discrete wavelet transform (DWT) hierarchically organizes data in a multi-resolution way by low and high pass filters. The low (high)-pass filters only pass low (high)-frequency signals but attenuate signals with frequencies higher (lower) than a cutoff frequency. As such, the DWT coefficients at the coarse level capture the global features of the input data and the coefficients at the fine levels capture the local features of the data, i.e., the low frequency and high frequency signals are represented by the coefficients in the coarse and fine resolutions respectively. Obviously, we can overcome the global feature selection mechanism after such a multi-resolution feature separation by selectively extracting local features and filtering redundant global features.

In this study, we present a novel multi-resolution independent component analysis (MICA) algorithm for effective feature selections for mass spectral data. Unlike the traditional feature selection methods, it suppresses redundant global features and extracts local features to capture gross and subtle data characteristics via multi-resolution data analysis. Then, we propose a multi-resolution independent component analysis based support vector machines (MICA-SVM) to achieve a high-performance proteomic pattern classification. In addition to rigorous machine learning analysis, we demonstrate the proposed classifier’s superiority by comparing it with nine state-of-the-art peers on six heterogeneous profiles generated from different profiling technologies and processed by different preprocessing algorithms. The exceptional classification performance (~99.5% average classification ratios) and excellent stability suggest this algorithm a great potential to facilitate mass spectral proteomics into a clinical routine, even if data reproducibility is not guaranteed.

## Methods

Multi-resolution independent component analysis (MICA) is built from the discrete wavelet transforms (DWT), principal component analysis (PCA), the first loading vector based data reconstruction, inverse discrete wavelet transforms (IDWT) induced meta-data approximation, and independent component analysis (ICA) based subspace spanning. The DWT decomposes input data in a multi-resolution form by using a wavelet and scaling function. Mathematically, it is equivalent to multiplying input data by a set of orthogonal matrices block by block. The coefficients at the coarse and fine levels represent input data’s global and local features respectively. Alternatively, ICA seeks to represent input data as a linear combination of a set of statistically independent components by minimizing their mutual information. Theoretically, it is equivalent to inverting the central limit theorem (CLT) by searching maximally non-normal projections of the original data distribution. More detailed information about DWT, PCA, and ICA can be found in [15,11].

### Multi-resolution independent component analysis (MICA)

MICA seeks the low dimensional meta-sample (prototype) for each high-dimensional mass spectral sample in the subspace generated by the statistically independent components from a meta-profile of the input data. As the same dimensional approximation of the original high-dimensional data, the meta-profile keeps the most important global features, drops the redundant global features, and exacts almost all local features of the original data. The meta-profile is computed by conducting an inverse DWT for the updated coefficient matrices, where the coarse level coefficients are selectively suppressed by the first loading vector reconstruction to filter the redundant global features, and the fine level coefficients are kept to extract the local features. It is worth pointing out that the independent components in MICA are calculated by conducting independent component analysis for the meta-profile. Unlike the independent components in the classic ICA that are mainly retrieved from the global features, the independent components calculated by MICA are statistically independent signals that contain contributions from almost all local features and the most important global features. As such, the latter is more representative in revealing the latent data structure than the former. Moreover, MICA brings an automatic de-noising mechanism via its redundant global feature suppressing. Since the coarse level coefficients (e.g., the first level coefficients) in the DWT generally contain “contributions” from noise, suppressing the coarse level coefficients not only filters unnecessary global features, but also removes the noise automatically. The automatic de-noising prevents noise from entering feature selection and the following classifier training, which will contribute to the robust mass spectral pattern classification. The MICA algorithm can be described as following steps.

### Algorithm 1 multi-resolution independent component analysis (MICA)

**1. Wavelet transforms.** Given a protein expression profile with *p* samples across *n* m/z ratios  MICA conducts a *L*-level column-wise DWT for input data to obtain wavelet coefficients, which consist of total *L* detail coefficient matrices:  and an approximation coefficient matrix  i.e.,  where 

**2. Redundant global feature suppressing and local feature extraction.** A level threshold  is selected to suppress redundant global features and maintain local features.

a). If 

1). conduct principal component analysis for each detail coefficient matrix *D_j_* to obtain its principal component (PC) matrix  and corresponding score matrix 

2). reconstruct and update the detail coefficient matrix *D_j_* by using the first loading vector *u*_1_ in the PC matrix as  where  is a *n_j_* × 1 vector with all entries being ‘1’s.

b). If *j* >*τ* keep all detail coefficient matrices  intact.

3). **Inverse discrete wavelet transforms.** Conduct the corresponding inverse discrete wavelet transforms using the updated coefficient matrices  to get the meta-profile of  i.e., 

4). **Independent component analysis**. Conduct the classic independent component analysis for X^*^ to obtain components and the mixing matrix: X^*^ = *AZ*^,^ where 

5). **Subspace decomposition.** The meta-profile X^*^ is the approximation of *X* by removing the redundant global features and retaining almost all local features by selecting features on behalf of their frequencies. It is easy to decompose each sample in the subspace spanned by all independent components  Each statistically independent component is a basis in the subspace, i.e.,  where the mixing matrix  and  In other words, each sample can be represented as  where the meta-sample *a_i_* is the *i^th^* row of the mixing matrix recording the coordinate values of the sample *x_i_* in the subspace. As a low dimensional vector, the meta-sample *a_i_* retains almost all local features and the most important global features of the original high-dimensional sample *x_i_*. Thus, it can be viewed as a data-locality preserved prototype of *x_i_*. It is worthwhile to note that each meta-sample in the subspace is the data locality persevered prototype of its corresponding high-dimensional mass spectral sample.

**Figure 1:**
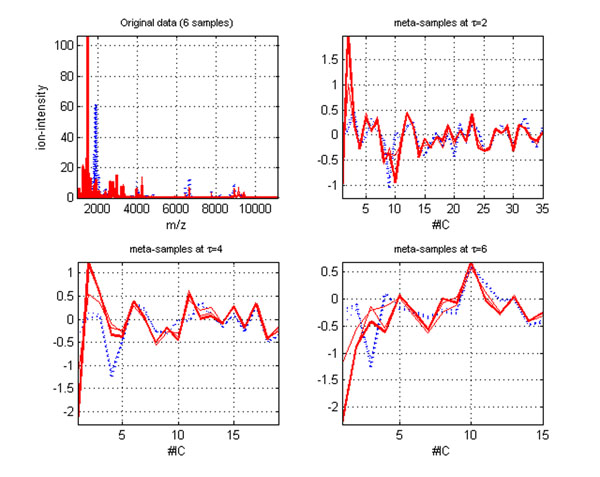
**Meta-samples computed from MICA.** Meta-samples computed from MICA for six original samples (three controls and three cancers) in the colorectal data at the three levels thresholds: τ=2,4,6 with the wavelet ‘*db8*’. The low-dimensional meta-samples separate two types of samples clearly in visualization (x-axis represents the dimensionality of the subspace spanned by the independent components).

**Figure 2:**
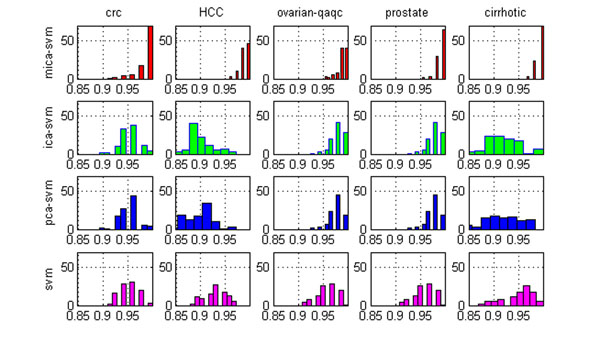
**Comparison of four SVM algorithms’ classification rate distributions under the 100 trials of 50% HOCV.** The distributions of the classification rates for the MICA-SVM, ICA-SVM, PCA-SVM and SVM algorithms on the five mass spectral datasets.

**Figure 3:**
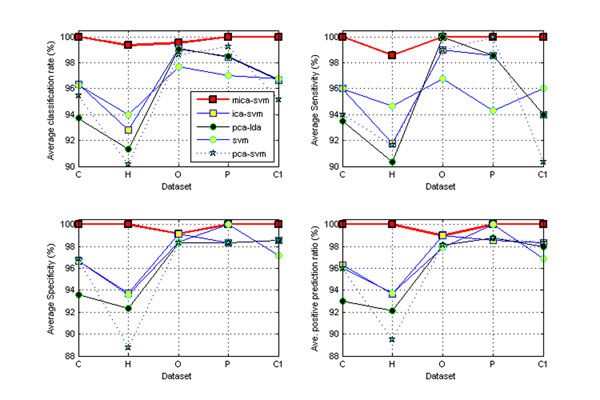
**Comparison of five algorithm performance under the 10-fold CV.** Comparisons of the classification performance of five algorithms under 10-fold CV on the five mass spectral profiles: ‘‘C’ (colorectal), ‘H’ (*hcc*) and ‘O’ (*ovarian-qaqc*), ‘P’ (*prostate*), and ‘C1’ (‘*cirrhotic*’). The MICA-SVM algorithm strongly demonstrates stably leading performance over the others.

The redundant global feature suppressing and local feature extraction in MICA decrease the total data variances for the following meta-profile by only keeping the data variance on the first PC of each coefficient matrix before or at the level threshold τ. As a same-dimensional but a low variance approximation for the original data by keeping the most important global data characteristics and capturing local data characteristics, the meta-profile X* makes the following independent component analysis more sensitive in catching subtle data behavior than applying ICA directly applying to the original data. Figure 1 visualizes three control and cancer samples of the colorectal (CRC) data [7]. Each sample is a 16331×1 vector, and their low-dimensional meta-samples are obtained from MICA at the thresholds τ=2,4,6 with a *Daubechies* family wavelet ‘*db8*’. We indicate the control and cancer samples and their corresponding meta-samples by *red* and *blue* lines respectively. It is clear that there is no any way to detect two types of samples from the plot of the original data (sub-fig 1 at the NW corner). However, their meta-samples at the three thresholds demonstrate clear separations between the controls and cancers (sub-fig 2,3,4 at the NE, SW, and SE corners). The extracted local features and selected important global features make two types of samples display two distinct prototypes in the low-dimension subspace. With the increase of the level thresholds, the two groups of prototypes tend to show more capabilities to separate cancer and control samples. Interestingly, two types of meta-samples demonstrate a “*self-clustering*” mechanism in that the meta-samples belonging to the same type show very close spatial proximities. Obviously, the clear sample separation information conveyed by the self-clustering mechanism of the meta-samples is almost impossible to obtain from the original high-dimensional data directly, and the key discriminative features captured by our proposed MICA method would be able to facilitate the subsequent classification step and contribute to high-accuracy disease diagnosis. It is also worth pointing out that similar results can be also obtained for the other mass spectral data.

### MICA-based support vector machines

The MICA-based support vector machine applies the classic support vector machine (SVM) [16] to the meta-samples calculated from MICA to gain classification in a low-dimensional space. Unlike the traditional SVM that builds a maximum margin hyperplane in the original high-dimensional space  where *n* ~ 10^3^ – 10^4^, MICA-SVM separates biological samples by constructing the maximum margin hyperplane in the spanned subspace  where  using the meta-samples. If we assume the number of support vectors *N_s_* is much less than the training points *l*, then, the time complexity of the MICA-SVM is  which is much lower than that of the classic SVM:  provided the same number of training points and support vectors. We briefly describe the MICA-SVM algorithm for binary classification at first.

Given a training dataset  and sample class type information  where  a meta-dataset  is computed by MICA. Then, a maximum margin hyper-plane:  is constructed to separate the '+1' (‘cancer’) and '-1' (‘control’) types of meta-samples. It is equivalent to solving the following quadratic programming problem,(1)

Eq. (1) can be solved through its Lagrangian dual that is also a quadratic programming problem, where  are the dual variables of primal variables *W* and *b*.(2)

The normal of the maximum-margin hyperplane is calculated as  and the intercept term *b* can be calculated as  The decision function  is used to determine the class type of a testing sample *x*', where  are the corresponding meta-samples of samples  computed from MICA respectively, and  is a SVM kernel function mapping the meta-samples into a same-dimensional or high-dimensional feature space. In this work, we mainly focus on the linear kernel for its efficiency in proteomic pattern classification. In fact, we have found that a SVM classifier under a standard Gaussian ( ‘*rbf*’) kernel kernel) inevitably encounters overfitting for mass spectral proteomic data through rigorously theoretical analysis. The details can be found in the additional file 1.

## Results

To demonstrate the superiority of our algorithm, we include five publicly available large-scale mass spectral profiles: colorectal (CRC) [7], hepatocellular carcinoma (HCC) [8], ovarian-qaqc, prostate [17], and cirrhotic [8], in our experiments. They are heterogeneous data generated from different profiling technologies and preprocessed by different algorithms. The HCC and cirrhotic datasets are two binary-class datasets separated from a three-class profile consisting of 78 HCC, 72 control, and 51 cirrhotic samples [8].

To address the data heterogeneity, we employed different preprocessing methods for these profiles. We conducted baseline correction, smoothing, normalization, and peak alignment for the *ovarian-qaqc* data. The baseline for each profile was estimated within multiple shifted windows of widths 200 m/z, and the spline approximation was applied to predict the varying baseline. The mass spectra were further smoothed using the ‘*lowess*’ method, and normalized by standardizing the area under the curve (AUC) to the group median. Moreover, the spectrograms were aligned to two reference peaks: (3883.766, 7766.166). Alternatively, we only conducted the baseline correction, normalization and smoothing for the HCC, prostate, and cirrhotic data, where the smoothing method was selected as the ‘*least-square polynomial’* smoothing instead of the ‘*lowess*’ smoothing. We did not conduct our own preprocessing for the colorectal data because it was preprocessed data [7]. Table 1 shows detailed information about the five data sets.

**Table 1:** Five heterogeneous mass spectral profiles

Dataset	#m/z	#Sample	Technology
Colorectal	16331	48 controls + 64 cancers	MALDI-TOF high resolution
HCC	23846	72 controls + 78 cancers	MALDI-TOF high resolution
Ovarian-qaqc	15000	95 controls + 121 cancers	SELDI-TOF high resolution
Prostate	15154	63 controls + 69 cancers	SELDI-TOF low resolution
Cirrhotic	23846	72 controls + 51 diseases	MALDI-TOF high resolution

### Cross validations and comparison peers

We compared our algorithm with six state-of-the-art peers in terms of average classification rates, sensitivities, and specificities under *k*-fold (*k*=10) and 100-trial of 50% holdout cross validations (HOCV). The classification accuracy in the *i^th^* classification is the ratio of the correctly classified testing samples over total testing samples:  The sensitivity and specificity are defined as the ratios:  respectively, where *tp* (*tn*) is the number of positive (negative) targets correctly classified, and *fp* (*fn*) is the number of negative (positive) targets incorrectly classified respectively. In the 100-trial of 50% holdout cross validation, all samples in each data set are pooled together and randomly divided into half to get training and testing data. Such a partition is repeated 100 times to get 100 sets of training and testing data sets. In the *k*-fold cross validation, an input dataset is partitioned into *k* disjoint, equal or approximately equal proportions. One proportion is used for testing and the other *k-1* proportions are used for training alternatively in the total *k* rounds of classifications. These cross validations are able to decrease potential biases in algorithm performance evaluations compared with the pre-specifying training or testing data approach.

The six comparison algorithms can be categorized into two types. The first type consists of the standard support vector machines (SVM) and linear discriminant analysis (LDA), both of which are the state-of-the-art classification methods. The second type consists of four methods embedding subspace feature-selections in SVM and LDA: they are support vector machines with principal component analysis (PCA) / independent component analysis (ICA) / nonnegative matrix factorization (NMF), and linear discriminant analysis (LDA) with principal component analysis. We refer to them as PCA-SVM, ICA-SVM, NMF-SVM, and PCA-LDA respectively. The implementation details of these algorithms can be found in [14].

### Experimental results

We employ the ‘*db8’* wavelet in MICA to conduct a 12-level discrete wavelet transform for each dataset and select the level threshold as τ=2 for all profiles uniformly. Although not an optimal level threshold for all data, it guarantees automatic de-noising and “*fair*” algorithm comparisons. Moreover, the meta-samples obtained from MICA at τ=2 can clearly distinguish two types of samples. Although other level threshold selections may be possible, any too ‘*coarse*’ (e.g.τ=1) or too ‘*fine*’ (e.g.τ=10) level threshold selection may miss some important global or local features and affect following classifications.

Table 2 and Table 3 illustrate the average performance of MICA-SVM and its six peers in terms of classification rates, sensitivities, specificities and their standard deviations under two types of cross validations respectively. The NMF-SVM and LDA algorithms are excluded from Table 3 for their relatively low performance. The best performance is highlighted for each data set. It is clear that the MICA-SVM algorithm achieved exceptionally leading advantages over the others. For example, the average prediction ratios attain >99.0% for all data under the 100 trials of 50% HOCV. It is interesting to see that our results are superior to those of the peak-selection based biomarker discovery methods. For instance, the peak-selection method employed by Alexandrov *et al *[7] achieved the SVM classification rate: 97.3% (sensitivity: 98.4% and specificity: 95.8%) on the colorectal (CRC) data under a double cross validation (a leave-one-out CV and 5-fold CV). Alternatively, another peak-selection biomarker discovery method induced by nonnegative principal component analysis (NPCA) attained 98.21% (sensitivity: 95.83% specificity: 100%) under a SVM classifier with the leave-one-out cross validation (LOOCV) on the same data set [14].

**Table 2:** Performance of seven algorithms under the 100 trials of 50% HOCV

Dataset	Ave. classification rate ± std (%)	Ave. sensitivity ± std (%)	Ave. specificity ± std (%)
**Colorectal**			
*mica-svm*	**99.05±01.82**	**98.84±03.41**	**99.28±01.82**
*svm*	95.71±02.01	95.28±03.67	96.19±02.88
*pca-svm*	95.37±01.98	93.60±04.18	96.83±02.93
*ica-svm*	95.57±02.02	93.69±04.11	97.11±02.81
*nmf-svm*	92.46±02.97	89.91±06.92	94.65±04.04
*lda*	87.39±04.60	84.97±07.56	89.36±06.11
*pca-lda*	94.21±02.75	93.87±03.64	94.51±04.03
**HCC**			
*mica-svm*	**99.07±01.03**	**98.82±01.73**	**99.31±01.62**
*svm*	93.08±02.33	93.42±03.55	92.95±04.12
*pca-svm*	89.65±02.86	89.09±04.46	90.33±04.59
*ica-svm*	90.15±02.63	89.76±04.27	90.70±04.35
*nmf-svm*	89.81±03.17	87.68±07.28	92.22±05.14
*lda*	89.48±03.67	91.55±04.42	87.75±06.88
*pca-lda*	91.20±02.81	90.08±05.18	92.38±03.62
**Ovarian-qaqc**			
*mica-svm*	**99.09±01.09**	**98.94±02.15**	**99.25±01.11**
*svm*	97.64±01.36	97.42±02.04	97.86±02.18
*pca-svm*	98.63±00.88	99.28±01.20	98.12±01.58
*ica-svm*	98.52±00.83	99.06±01.30	98.10±01.50
*nmf-svm*	92.47±03.23	94.23±03.72	91.15±04.89
*lda*	81.42±04.48	87.86±05.17	76.26±06.91
*pca-lda*	98.42±01.04	99.30±01.13	97.73±01.94
**Prostate**			
*mica-svm*	**99.36±00.99**	**99.09±01.43**	**99.64±01.66**
*svm*	95.91±02.09	95.75±03.05	96.18±03.99
*pca-svm*	97.94±01.65	98.48±01.70	97.43±03.24
*ica-svm*	98.23±01.61	98.36±01.84	98.14±02.84
*nmf-svm*	91.21±04.67	94.44±04.70	87.46±06.69
*lda*	89.92±04.77	94.43±04.65	85.02±10.46
*pca-lda*	97.50±02.20	97.90±02.50	97.10±03.25
**Cirrhotic**			
*mica-svm*	**99.52±00.85**	**99.44±01.65**	**99.62±00.95**
*svm*	95.10**±**03.17	92.97**±**05.91	96.71**±**03.10
*pca-svm*	91.52**±**03.76	88.00**±**08.39	94.15**±**03.86
*ica-svm*	92.07**±**03.41	88.47**±**07.92	94.72**±**03.48
*nmf-svm*	88.03±03.10	80.43±07.84	93.42±03.62
*lda*	86.66±06.11	86.57±10.30	86.93±08.19
*pca-lda*	92.39±03.62	89.04±07.45	94.83±03.48

**Table 3:** Five classifier performance under the 10-fold CV

Dataset	Ave. classification rate ± std (%)	Ave. sensitivity ± std (%)	Ave. specificity ± std (%)
**Colorectal**			
*mica-svm*	**100.0 ± 00.00**	**100.0 ± 00.00**	**100.0 ± 00.00**
*pca-lda*	93.71 ± 07.46	93.50 ± 10.55	93.57 ± 11.45
*svm*	96.27 ± 06.45	96.00 ± 08.43	96.67 ± 07.03
*pca-svm*	95.45 ± 06.43	94.00 ± 09.66	96.90 ± 06.55
*ica-svm*	96.35 ± 04.73	96.00 ± 08.43	96.67 ± 07.03
**HCC**			
*mica-svm*	**99.33 ± 02.11**	**98.57 ± 04.52**	**100.0 ± 00.00**
*pca-lda*	91.33 ± 05.49	90.36 ± 06.69	92.32 ± 08.87
*svm*	93.99 ± 06.55	94.64 ± 09.11	93.57 ± 09.00
*pca-svm*	90.16 ± 06.20	91.61 ± 09.89	88.75 ± 10.94
*ica-svm*	92.79 ± 07.10	91.79 ± 11.42	93.75 ± 08.84
**Ovarian-qaqc**			
*mica-svm*	**99.52 ± 01.51**	**100.0 ± 00.00**	**99.17 ± 02.64**
*pca-lda*	99.07 ± 01.96	100.0 ± 00.00	98.33 ± 03.51
*svm*	97.68 ± 03.25	96.78 ± 05.20	98.40 ± 03.38
*pca-svm*	98.61 ± 02.23	99.00 ± 03.16	98.33 ± 03.51
*ica-svm*	99.09 ± 01.92	99.00 ± 03.16	99.17 ± 02.64
**Prostate**			
*mica-svm*	**100.0 ± 00.00**	**100.0 ± 00.00**	**100.0 ± 00.00**
*pca-lda*	98.52 **±** 03.13	98.57 **±** 04.52	98.33 **±** 05.27
*svm*	96.98 ± 03.90	94.29 ± 07.38	100.0 ± 00.00
*pca-svm*	99.23 ± 02.43	100.0 ± 00.00	98.33 ± 05.27
*ica-svm*	98.45 ± 03.27	98.57 ± 04.52	98.33 ± 05.27
**Cirrhotic**			
*mica-svm*	**100.0 ± 00.00**	**100.0 ± 00.00**	**100.0 ± 00.00**
*pca-lda*	96.73 ± 05.77	94.00 ± 09.66	98.57 ± 04.52
*svm*	96.79 **±** 04.14	96.00 ± 08.43	97.14 ± 06.02
*pca-svm*	95.13 ± 05.75	90.33 ± 13.92	98.57 ± 04.52
*ica-svm*	96.67 ± 05.83	94.00 ± 13.50	98.57 ± 04.52

However, our algorithm achieved the average 99.05% classification rate (sensitivity: 98.84% and specificity: 99.28%) under 100 trials of 50% HOCV where much less priori knowledge are available in classification than the LOOCV and 5-fold cross validation. In addition, under the 10-fold cross validation, the proposed algorithm achieves 99.33% and 99.52% predication ratios on the HCC and ovarian-qaqc data respectively. More impressively, it attains 100% classification ratios on the colorectal, prostate, and cirrhotic data. Unlike the other methods displaying instabilities in classifications, our algorithm demonstrates strong stability in attaining high-accuracy pattern detections for all the five profiles. This observation is also supported by its lower standard deviations of the three classification measures of MICA-SVM than those of the others.

We also have found that there are almost no statistically significant differences between SVM and its subspace feature selection based extensions (e.g., PCA-SVM), which achieve same level or slightly lower performance than the standard SVM. The reason seems to be rooted in the global feature selection mechanisms of the PCA, ICA, and NMF methods. As we pointed out before, since some mass spectral samples may display very similar global-characteristics but different local-characteristics, a SVM classifier integrated with a global feature selection method may inevitably encounter difficulty in distinguishing these samples. Although extracted by different transformation methods, the global features seem to have nearly same level contributions to proteomic data classification statistically. Moreover, the redundant global features brought by the global feature selection mechanism may get involved in the SVM learning, which would limit all the SVM-related classifiers’ generalization and cause instability in classification. This point can be also observed through their relatively high standard deviations of the classification rates, sensitivities and specificities. For example, the standard deviations of the three measures from the PCA-SVM classifier are 3.76%, 8.39%, and 3.86% respectively, which are much higher than those from the MICA-SVM classifier (0.85%, 1.65%, and 0.95%) on the cirrhotic profile. Similar observations can also be found for the other data sets.

However, it is interesting that MICA’s local feature capturing and redundant global feature suppressing mechanism appear to contribute to the MICA-SVM classifier’s exceptional performance and good algorithm stability on the five heterogeneous data sets. Figure 2 compares the distribution of the MICA-SVM classifier’s classification rates with those of the ICA-SVM, PCA-SVM and SVM classifiers under the 100 trials of 50% HOCV. It clearly demonstrates that MICA-SVM has statistically significant advantages over the other three classifiers on all five data sets. Moreover, Figure 3 shows MICA-SVM’s leading advantages over its four peers: PCA-LDA, PCA-SVM, ICA-SVM, and SVM, in terms of the average classification rates, sensitivities, specificities, and positive prediction ratios under the 10-fold CV. Consistent to the cases in the 100 trials of 50% HOCV, the four peers also show a nearly same level performance on the four classification measures.

### Multi-class classification

The MICA-based support vector machines can be also extended to handle the multi-class classification, which has not been seriously addressed in mass spectral proteomics. However, it can be more practical in cancer diagnosis because detecting different pathologic states of cancers is essential in early cancer discovery. We ‘merge’ the HCC and cirrhotic data into a three-class profile to seek high-accuracy detection between healthy individuals (controls) and patients with hepatocellular carcinoma (HCC) and cirrhosis, where cirrhosis can be viewed as an early HCC stage to some degree because chronic hepatitis C causes HCC via the stage of cirrhosis.

We employ the ‘*one-against-one’* method in our MICA-based multi-class SVM classification for its proved advantage over the ‘*one-against-all’* and ‘*directed acyclic SVM’* methods [18]. The ‘*one-against-one’* method builds *k*(*k-1*)*/2* binary SVM classifiers for a data set with *k* classes: *{1*,*2*,*…k}*. Each classifier is trained on data from two classes, i.e., training samples are from the *i*th and *j*th classes where *i*,*j*=*1*,*2*,*..k*. We describe our MICA-based ‘*one-against-one’* SVM as follows.

Given a training data set consisting of  samples across *m* testing points from the *i*th and *j*th classes i.e.,  and their corresponding labels  a corresponding low dimensional meta-sample data  is computed by MICA. Then, maximizing the margin between two types of data is equivalent to the following problem:(3)

where *a_t_* is the meta-sample calculated for the training sample *x_i_*. After building all *k*(*k-1*)*/2* classifiers, we first determine if a testing sample *x*' is from class the *i*th or *j*th class by a local decision function  where *a*' is the meta-sample of *x*'. Then, we use the ‘*Max-wins’* voting approach to infer its final class type: if the local decision function says *x*' is in the *i*th class, then the *i*th class wins one vote; Otherwise, the *j*th class wins one vote. Finally, *x*' will belong to the class with the largest vote.

We also implemented the ‘*one-against-one’* method in SVM, PCA-SVM and ICA-SVM multi-class classification for a fair comparison. It was interesting to find that the four classifiers: PCA-LDA, SVM, PCA-SVM, and ICA-SVM had equivalent performance under the two types of cross validations for this trinary data. Just as before, the LDA and NMF-SVM algorithms had lower level performance than those of the four algorithms. However, the MICA-SVM algorithm achieved average classification ratios: 97.37% and 98.52% respectively under the 100 trials of 50% HOCV and 10-fold CV, which were much higher than the corresponding average 83.79% and 86.61% level classification ratios attained by the four peers under the same cross validations.

**Figure 4:**
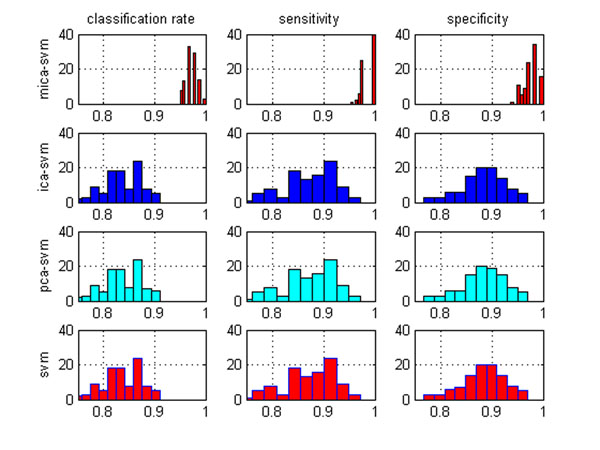
**Multi-class classification performance**. The distributions of the classification rates, sensitivities and specificities of the MICA-SVM, ICA-SVM, PCA-SVM and SVM algorithms on a three-class data set. The distributions of the three random variables: classification rates, sensitivities and specificities of the MICA-SVM algorithm are significantly different from those of the other three algorithms for its exceptional classification performance.

Figure 4 compares the classification performance of our proposed algorithm with those of the PCA-SVM, ICA-SVM and SVM algorithms under the 100 trials of 50% HOCV by visualizing the distributions of their classification rates, sensitivities, and specificities. The similar or even identical distributions of the three random variables suggest there are no statistically significant differences between the three classifiers. However, the distributions of the three random variables for the MICA-SVM algorithm imply it is significantly different from those comparison algorithms by attaining high-accuracy pattern prediction. On the other hand, it appears that that integrating an ‘*one-against-one*’ SVM with the global feature selection algorithms (e.g., PCA, ICA) may not contribute to enhancing multi-class data classification either. However, integrating the ‘*one-against-one*’ SVM with MICA demonstrates a statistically significant improvement in multi-class classification for its effective local feature capturing. Such results are also consistent to those of the previous binary classification.

### MICA-based linear discriminant analysis

Although linear discriminant analysis (LDA) had the worst performance among all seven algorithms in our investigation, it would be interesting to generalize MICA to LDA classification by designing a MICA-LDA classifier to further verify the effectiveness of MICA in enhancing proteomic pattern detection, and take advantage of LDA’s built-in multi-class handling mechanism. Similar to the MICA-SVM algorithm, the multi-resolution independent component analysis based linear discriminant analysis (MICA-LDA) applies the classic LDA to the meta-samples obtained from MICA to gain sample classification. Table 4 shows the MICA-LDA algorithm’s performance on the six profiles. To keep consistency with the previous experiments, we still use the ‘*db8*’ wavelet and set the level threshold τ=2 in MICA. Interestingly, this algorithm’s performance is only secondary to that of the MICA-SVM algorithm. It achieves a 96.84% average classification rate with 98.69% sensitivity and 96.21% specificity on the three-class profile under the 100 trials of 50% HOCV. Furthermore, it outperforms the other comparison algorithms on the colorectal, cirrhotic, and HCC data.

**Table 4:** MICA-LDA performance on six mass spectral data sets

Cross validation	Ave. classification rate ± std (%)	Ave. sensitivity ± std (%)	Ave. specificity ± std (%)
**50%HOCV**			
*Colorectal*	96.21±02.07	96.20±03.70	96.29±02.53
*HCC*	98.19±01.67	100.0±00.00	96.50±03.21
*Ovarian-qaqc*	90.62±02.73	91.93±04.67	89.58±04.05
*Prostate*	93.03±03.05	96.15±03.24	89.42±05.24
*Cirrhotic*	98.93±01.37	97.52±03.05	99.97±00.28
*Three-class*	96.84±01.32	98.69±01.49	96.21±02.10
**10-fold CV**			
*Colorectal*	96.26 ± 06.77	95.00 ± 15.81	96.90 ± 06.55
*HCC*	97.38 ± 03.39	100.0 ± 00.00	95.00 ± 06.45
*Ovarian-qaqc*	88.46 ± 07.16	91.78 ± 13.41	86.03 ± 06.54
*Prostate*	90.87 ± 07.86	92.86 ± 10.10	88.57 ± 13.65
*Cirrhotic*	99.17 ± 02.64	98.00 ± 06.32	100.0 ± 00.00
*Three-class*	97.33 ± 03.44	96.25 ± 06.04	98.57 ± 04.52

### Three partial least square (PLS) based regression methods

We also compare our algorithm with three PLS-based regression methods. As an interesting dimension reduction algorithm originally developed in the field of chemometrics, PLS recently draws more and more attention in machine learning and statistical inference. The three PLS-based regression methods consist of the PLS-based regression, PLS-based linear logistic regression proposed by Nguyen and Roche [19], and PLS-based ridge penalized logistic regression proposed by Fort and Lambert-Lacroix [20]. In our context, all the three algorithms treat classification as a regression one with discrete outputs under few observations and many predictor variables. We refer to them as PLS-REG, NR-LLD, and RPLS-LLD respectively. Since the NR-LLD and RPLS-LLD algorithms require feature selection before classification, we conduct a two-sample t-test with pooled variance estimate to select the 2000 most differentially expressed features from each data set for the two methods, where the three-class data set is treated as a binary data set with 72 controls and 129 diseased samples (78 hepatocellular carcinoma +51 cirrhosis samples). The number of PLS components are uniformly selected as 10 for all the three methods. Table 5 shows MICA-SVM and the three algorithms’ average classification rates and their standard deviations from the two types of cross validations. It is interesting to see that our proposed MICA-SVM algorithm still hold obvious advantages over the three peers in performance.

**Table 5:** Performance of MICA-SVM, PLS-REG, NR-LLD, and RPLS-LLD

Algorithms	MICA-SVM	PLS-REG	NR-LLD	RPLS-LLD
Data	Average classification rates under the 100 trials of 50% HOCV (%)			

*Colorectal*	99.05±01.82	96.64±02.19	97.02±01.78	96.23±02.32
*HCC*	99.07±01.03	94.60±02.12	91.09±02.65	94.40±02.19
*Ovarian-qaqc*	99.09±01.09	95.44±02.00	98.18±01.52	96.68±02.00
*Prostate*	99.36±00.99	98.32±01.43	96.74±01.97	98.32±01.47
*Cirrhotic*	99.52±00.85	91.36±03.44	89.82±03.50	92.84±02.36
*Three-class*	97.37±01.20	68.51±04.82	84.04±03.66	85.23±03.31

Data	Average classification rates under10-fold CV (%)			

*Colorectal*	100.0±00.00	94.53±08.80	98.09±04.03	97.18±06.25
*HCC*	99.33±02.11	92.70±07.26	93.32±07.26	93.37±06.29
*Ovarian-qaqc*	99.52±01.51	98.64±04.31	98.18±03.18	98.16±02.38
*Prostate*	100.0±00.00	99.32±02.43	96.26±05.19	99.29±02.26
*Cirrhotic*	100.0±00.00	95.06±07.00	89.36±09.60	94.36±06.62
*Three-class*	98.52±03.35	77.11±08.23	85.99±08.17	88.64±06.83

### Algorithmic stability analysis

The instabilities of current classification methodologies are widely found in mass spectral proteomics. In fact, almost all of these classification methods were proposed through analyzing an individual dataset [1-3,5,7,8]. They may work efficiently on the individual data but lack stability when applied to other heterogeneous data generated from different profiling technologies or processed by different preprocessing methods. In fact, such instabilities not only present difficulties in reproducible biomarker discovery, but also hamper exploring the clinical potentials of this technology. Although algorithmic stability analysis is essential in computational proteomics, there is even no ad-hoc investigation on this topic. To evaluate the algorithmic stabilities of mass spectral proteomic data classification algorithms, we present an algorithmic stability analysis by introducing two scale-free measures: algorithm stability index and relative stability. The algorithm stability index measures the stability of an algorithm across a number of datasets. A high algorithm index value indicates better stability of an algorithm. Alternatively, the relative stability measures the stabilities of a set of classification algorithms with respect to a specific algorithm, which is selected as the MICA-SVM algorithm in this study. A small relative stability indicates an algorithm with a relatively close performance to that of the MICA-SVM algorithm.

Given a classification algorithm running on *M* heterogeneous profiles under a cross validation, the algorithm stability index *δ_a_* and the relative stability *δ_r_* are defined as,  where *μ_i_*, *s_i_* are the average classification rate and the corresponding standard deviation of the algorithm on the *i^th^* profile respectively, and the parameter  is the average classification ratio of the MICA-SVM algorithm on the *i^th^* profile.

**Figure 5:**
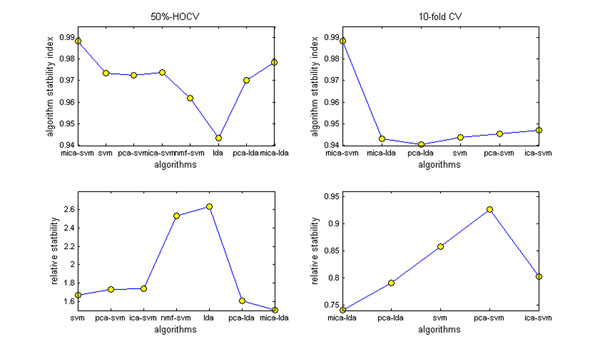
**Algorithmic stability analysis**. The algorithm stability index and relative stability values under the 100 trials of 50% HOCV and 10-fold CV. The MICA-SVM algorithm has the largest stability among all eight algorithms, and MICA-LDA has the closest performance to that of the MICA-SVM algorithm.

The two left figures in Figure 5 show the algorithm stability index and relative algorithm stability values of all eight algorithms on the six profiles under the 100 trials of 50% HOCV. It is interesting to see that the PCA-SVM, ICA-SVM, and SVM algorithms have almost same level stabilities for their close *δ_a_* values. The two smallest *δ_a_* values suggest the least stabilities of the NMF-SVM and LDA algorithms. The *δ_a_* values of the MICA-SVM and MICA-LDA algorithms are the largest and 2^nd^ largest among the eight algorithm index values. The relative stability value of the MICA-LDA algorithm suggests it achieve the closest performance with respect to the MICA-SVM algorithm. At the same time, the two right figures in Figure 5 illustrate similar observations for the two measures on the six algorithms (The two least stable algorithms NMF-SVM and LDA are excluded) under the 10-fold CV. Obviously, the MICA-SVM algorithm still maintains its highest stability when more priori knowledge is available in classification. Although the relative stabilities of the PCA-SVM, ICA-SVM, SVM, PCA-LDA, and MICA-LDA algorithms have the same ‘*ordering*’ as those of the five methodologies under the 50% HOCV, all the five algorithms have smaller relative stability values because more prior knowledge is available in the classifications under the 10-fold CV.

### Optimal level threshold selection

A remaining question is how to determine the optimal level threshold in MICA so that the following SVM classifier achieves best performance. It is reasonable to believe an optimal level threshold will contribute to capturing important local and global features of the original data in the meta-samples. We here employ a log-condition number  of the mixing matrix *A* to estimate the status of global and local feature capturing, where *λ*_max_ and *λ*_min_ are the maximum and minimum singular values of the mixing matrix. A large log-condition number indicates the better global and local feature capturing. The level-threshold is counted ‘*optimal*’ if the log-condition number of the mixing matrix is the largest. If log-condition numbers from two level thresholds are same numerically, the lower level threshold (which is required to be > 1) is counted as the optimal one. For instance, the largest and 2^nd^ largest α values are achieved at τ=1 and τ=7 respectively on the ovarian-qaqc data. However, our algorithm achieved the best average classification performance at τ=7, where the average classification rate, sensitivity and specificity are 99.74%, 99.73% and 99.76% respectively (The average classification rate is 95.28% at τ=1).

**Figure 6:**
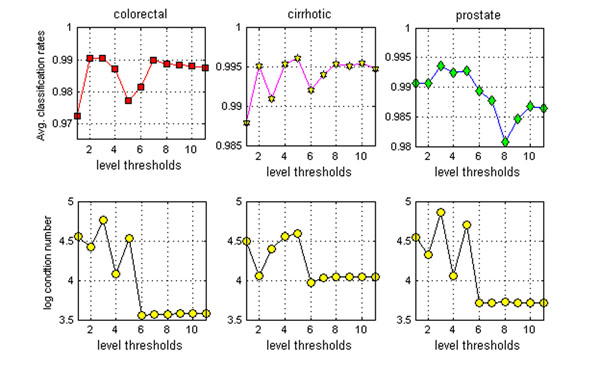
**Optimal level threshold selections.** Average classification rates and corresponding log-condition numbers at 11 level thresholds on the colorectal, cirrhotic and prostate data under the 100 trials of 50% HOCV.

Figure 6 shows the MICA-SVM average classification rates and corresponding α values under the 100 trials of 50% HOCV on the colorectal, cirrhotic, and prostate data, when the level threshold values are from 1 to 11 in MICA. It is interesting to see that the average classification rates have some or significant decreases when the level threshold values τ≥6 where the corresponding log-condition numbers show some level ‘stability’. However, it seems that the level threshold corresponding to the maximum log-condition number indicate the optimal or near optimal level classification performance in our experiment. Furthermore, we also have found that the MICA-SVM algorithm’s performance may decrease with too coarse level thresholds (e.g., τ =1) and too fine level thresholds (e.g., τ ≥8). Since the optimal level threshold selection method may increase computing complexities in classification for its maximum log-condition number computing. In practice, we suggest the empirical level threshold as *2≤τ≤L/3* for its robust performance and automatic de-noising property. In addition, we discuss possibly optimal wavelet selection for MICA-SVM under different cross validations, which can be found in the additional file 2.

## Discussion

In this study, we present a multi-resolution feature selection algorithm: multi-resolution independent component analysis (MICA) for effective feature selection for mass spectral data, propose a high-performance classification algorithm for heterogeneous proteomic profiles, and demonstrate its superiority by comparing it with nine peers. Our approach seeks reproducible high-accuracy diagnosis by treating an input profile a whole biomarker from a machine-learning viewpoint. It shows a great potential to facilitate mass spectral proteomics technology into a clinical routine, even if the data reproducibility is not guaranteed. It is worthwhile to note that independent component analysis is a necessary step to achieve good classification performance. We have found that a similar multi-resolution principal component analysis based SVM algorithm is not able to reach a comparable performance as our algorithm because of the loss of statistical independence in the feature selection. Although our methodology can achieve the clinical-level disease diagnosis for mass spectra even if the data reproducibility is not guaranteed, we do not intend to de-emphasize the importance in enhance mass spectral proteomic profile reproducibility because of its potential in identifying reproducible biomarkers. In fact, previous studies [21] pointed out that data reproducibility may affect data analysis and bring biases. For example, hierarchical clustering may bring different results for mass spectra acquired in day one and the same data a month later. However, it is also reasonable to expect the proposed algorithm’s exceptional performance on the mass spectral data with robust reproducibility for its generality on heterogeneous data.

## Conclusions

Our study suggests a new direction to accelerate mass spectral proteomic technologies into a clinical routine. The novel concepts of global and local feature selection, multi-resolution data analysis based redundant global feature suppressing, and effective local feature extraction techniques proposed in this study will also have positive impacts on large scale ‘*omics*’ data mining. The exceptional discriminative power demonstrated by MICA-based classifiers in multi-class proteomic data classification also contributes to early stage cancer diagnosis. It is interesting to find the MICA-based methods can be also applied to achieve exceptional gene expression pattern classification and meaningful biomarker discovery [22]. In the following work, in addition to further polishing our algorithm by comparing them with other state-of-the-art methodologies or data analysis tools [23], we are interested in investigating the multi-resolution independent component analysis based unsupervised or semi-supervised learning algorithms in proteomic pattern discovery by integrating the multi-resolution feature selection with the state-of-the-art clustering or semi-supervised learning algorithms, and generalize corresponding methods to the related topics such as gene subnetwork identification [24], and biomedical text classification in our future work.

## Competing interests

The author declares that there is no competing interest.

## Authors' contributions

HEY did all work for this paper

## Supplementary Material

Additional file 1 **Overfitting analysis** A rigorous analysis on SVM overfitting under a standard Gaussian kernel for mass spectral proteomic data. Click here for file

Additional file 2 Wavelet selection for MICA-SVM Click here for file
