# Supplementary material for: A high performance profile-biomarker diagnosis for mass spectral profiles
Source: BMC Syst Biol. 2011 Dec 14;5(Suppl 2):S5. doi: 10.1186/1752-0509-5-S2-S5 (PMC3287485; doi:10.1186/1752-0509-5-S2-S5)
Supplement: Additional file 1 — Overfitting analysis A rigorous analysis on SVM overfitting under a standard Gaussian kernel for mass spectral proteomic data. [file 1752-0509-5-S2-S5-S1.pdf]

Overfitting analysis

We only employ the '*linear*' kernel in the MICA-SVM algorithm. However, we found that this algorithm also encounters over-fitting (e.g., when τ=2,3) under a standard Gaussian ('*rbf*') kernel ($k(x,y)=\exp(-\|x-y\|^2/2)$ where *x*,*y* represent two samples) as the SVM, PCA-SVM, and ICA-SVM algorithms. Interestingly, *the over-fitting has its own special characteristics*: the SVM-based algorithms can only recognize the majority type of the training data in each classification trial because their corresponding kernel matrices are identity or near identity matrices. Since these algorithms have almost identical performance under the '*rbf*' kernel, without loss of generality, we use the SVM algorithm to address the special characteristics associated with over-fitting and prove over-fitting is actually inevitable for these learning machines on mass spectral data.

We show each SVM kernel matrix under the '*rbf*' kernel is identity or near identity. For convenience, we treat all samples in each mass spectral profile as training samples that includes all possible training entries for a SVM learning machine under any cross validations. The sub-figure 1 in the following Figure shows that the minimum and 1st percentile values of all possible square distances between the samples: $\|x_i - x_j\|^2, i \neq j$. By the definition of the '*rbf*' kernel, it is easy to find that any non-diagonal kernel entry is at least $< e^{-2^{-9}}$ for the colorectal, HCC, cirrhotic and prostate data and their kernel matrices are identity matrices. This point is also supported by the sums of all non-diagonal entries in their kernel matrices, i.e., $\sum_{i \neq j} k_{ij} = 0$ in the sub-fig 2. Although the kernel matrix of the ovarian-qaqc data is not an identity matrix, the small sum of its kernel non-diagonal entries: 1.386×10^{-5} indicates it is a near identity matrix, a matrix with approximately zero non-diagonal entries) because of only 156 non-diagonal entries $>10^{-15}$ (99.66% of them $\leq 10^{-15}$. The sub-figure 3 visualizes 78 non-diagonal items in the lower-triangle of its kernel matrix. Like an identity matrix, the kernel matrix has all eigenvalues equal to 1 as (sub-fig 4). Obviously, these identity or near identity kernel matrices can only represent the concept of identity and have no way to generalize to other new data in the SVM learning.

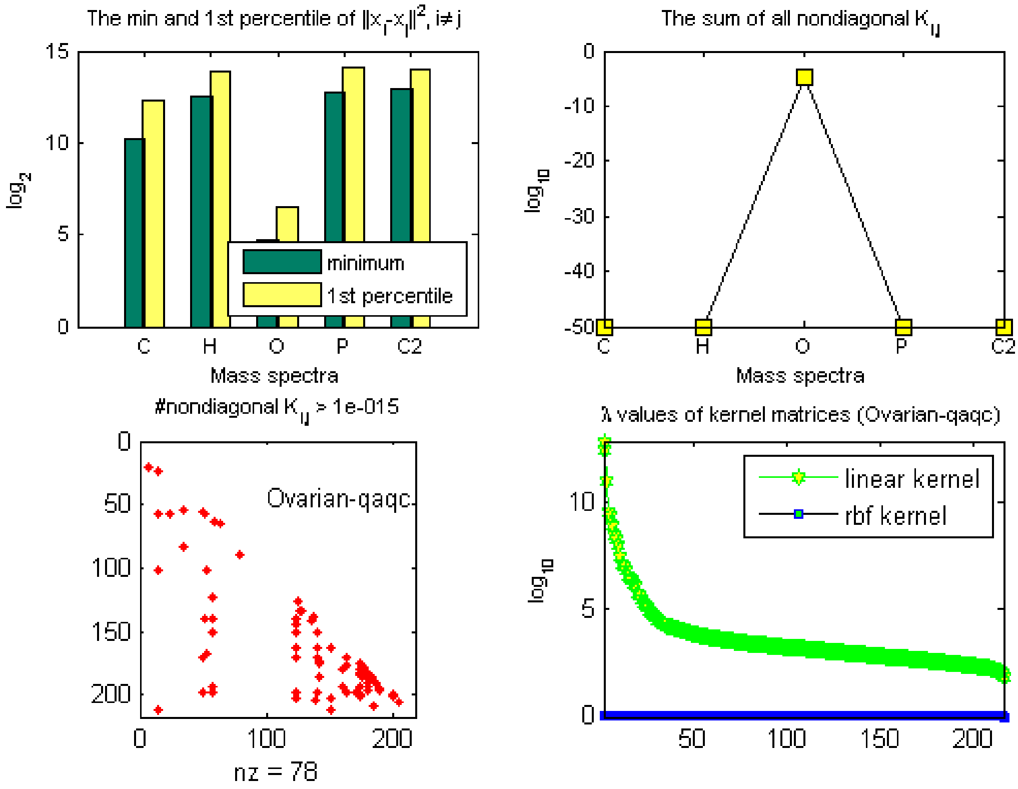

Fig S1. The minimum and 1st percentile values of all possible square distances between samples for all five profiles (sub-fig1), where each data set is represented by its first letter, i.e., ''C' (colorectal), 'H' (*hcc*) and 'O' (*ovarian-qaqc*), 'P' (*prostate*), and 'C2' ('*cirrhotic*'); The sums of all non-diagonal entries in each Gaussian kernel matrix (sub-fig2); Visualizations of the non-diagonal entries $>10^{-15}$ in the lower triangle Gaussian kernel matrix for the ovarian-qaqc data (sub-fig3); The eigenvalues of the linear and Gaussian kernel matrix of the ovarian data (sub-fig4).

The following theorem states that SVM can only recognize the majority type of a training data no matter the type of a test sample if its kernel matrix is the identity or near identity. A majority (non-majority) type simply refers to the type with more (less) counts in a binary-class profile. For example, 'cancer' is the majority type because it has 69 counts among all 112 colorectal samples. A majority type ratio is the number of majority type samples over the total samples (e.g. 69/112).

Theorem Let $X=[x_1,x_2,\cdots x_n]^T$, $x_i \in \mathbb{R}^m$ be a training dataset with n samples across m m/z ratios ($n \ll m$), drawn from a protein expression profile $D \in \mathbb{R}^{N\times m}$ ($N > n$), inputted to a standard SVM with a kernel function $k(x,y)$. The training sample labels are specified as $c=[c_1,c_2\cdots c_n]^T$, $c_i \in \{-1,1\}$, where '-1' and '1' represent the 'cancer' and 'control' classes respectively. Then for a testing sample $x' \in D-X$, its class type can be determined as

$$f(x') = \begin{cases} 1 & if\ \left|\{c_i \mid c_i = +1\}\right| > \left|\{c_i \mid c_i = -1\}\right| \\ -1 & if\ \left|\{c_i \mid c_i = -1\}\right| > \left|\{c_i \mid c_i = +1\}\right| \\ 0 & if\ \left|\{c_i \mid c_i = -1\}\right| = \left|\{c_i \mid c_i = +1\}\right| \end{cases} \qquad \text{(E1)}$$

Provided the kernel term is zero or approximately zero for a pair of samples $\forall x_i, x_j \in D, i \neq j$, $k(x_i, x_j) \sim 0$.

Proof. For a testing sample x', function $f(x') = sign(\sum_{i=1}^{n} \alpha_i c_i k(x_i \bullet x') + b)$ determines its class type where $\alpha_i \geq 0, i = 1, 2 \cdots n$, are the solutions of Eq (2), the dual problem of the QP problem induced by SVM.

$$\begin{aligned} &\max_{\alpha} J_D(\alpha) = -\frac{1}{2}\sum_{i=1}^{n}\sum_{j=1}^{n} c_i c_j k(x_i, x_j)\alpha_i \alpha_j + \sum_{i=1}^{n}\alpha_i \\ &s.t. \qquad \sum_{i=1}^{n}\alpha_i c_i = 0 \\ &\qquad\qquad 0 \leq \alpha_i \leq C, i = 1, 2 \cdots n \end{aligned} \qquad \text{(E2)}$$

When the kernel term is zero or approximately zero: $k(x_i, x') \sim 0$, the classification will only rely on the bias term *b:* $f(x') = sign(b)$, $b = -\frac{1}{2}(\sum_{j=1}^{n}\alpha_j c_j k(x_j, x_p) + \sum_{j=1}^{n}\alpha_j c_j k(x_j, x_n))$, where x_p and x_n are samples with '+1' and '-1' labels respectively. Since $k(x_j, x_p) \sim 0$, $k(x_j, x_n) \sim 0$, we have $b = -\frac{1}{2}(\alpha_p - \alpha_n)$ where α_p and α_n are alpha values for a positive sample and negative sample respectively. Alternatively, since $k(x_i, x_j) \sim 0$ at $i \neq j$, the problem in Eq. (E2) is simplified as a trivial problem with the objective function: $J_D(\alpha) = -\frac{1}{2}\sum_{i=1}^{n}\alpha_i^2 + \sum_{i=1}^{n}\alpha_i$

There are only two different alpha values in the trivial problem's solution: $\alpha_1 = \alpha_2 = \cdots = \alpha_{l_1} = l_2 / n,\ \alpha_{l_1+1} = \alpha_{l_1+2} = \cdots = \alpha_{l_1+l_2} = l_1 / n,$ where l_1 and l_2 are cardinalities of sets of '+1' and '-1' samples respectively, i.e., $l_1 = |\{c_i \mid c_i = +1\}|$, $l_2 = |\{c_i \mid c_i = -1\}|$, $l_1 + l_2 = n.$ For convenience, we denote $\alpha_p = l_2 / n,\ \alpha_n = l_1 / n.$ Correspondingly, the decision function for an input sample x' is simplified as $f(x') = sign(\frac{l_1}{2n} - \frac{l_2}{2n}).$ Obviously, the class type of x' will be totally determined by which type is the majority type in the training data, i.e., $l_1 > l_2$ or $l_2 > l_1$. If there is no majority type in the dataset, i.e., $l_1 = l_2$, the learning machine cannot determine the class type of the input sample x' and the classification rate will be zero.

Since a SVM learning machine can only recognize the majority type of the training data under the '*rbf*' kernel according to the theorem, it is interesting to see that the classification ratios for the five profiles under the 10-fold CV are just their majority type ratios: 64/112 (colorectal), 78/150 (HCC), 121/216 (ovarian-qaqc), 69/132 (prostate), 78/123 (cirrhotic), and their corresponding specificities or sensitivities are 100% or 0% respectively. However, the average classification rate for each profile under the 100 trials of 50% HOCV is approximately its majority type ratio because some non-majority type may work as 'local majority type' due to sampling. However, the corresponding average sensitivity and specificity are still complementary to each other. For example, the average classification rate for the colorectal data is 56.21% <57.14% (64/112) with sensitivity: 2% and specificity: 98%, which means the non-majority type ('control') was counted as the 'local majority type' in 2 trials among the 100 trials of classifications. If #majority-type is close to #non-majority-type, then the likelihood that a non-majority type is counted as 'local majority type' in a training data will be high. Correspondingly, the average classification rate will move far from the majority-type ratio. For example, the prostate data has 69 majority-type ('cancer') samples and 63 non-majority-type ('control') samples. The average classification rate for this data is 47.86%, which is far from majority-type ratio 52.27% (69/132), with sensitivity 67% and specificity 33%, i.e., the non-majority type is counted as the 'local majority type' in 33 trials among the 100 trials of classifications.

The biological reason for the over-fitting problem associated with the SVM-based classification is the sensitive signal-amplification mechanism from mass spectral proteomics technologies, where any subtle changes in the part of proteome will be amplified to large differences in mass spectral expressions. The exponential transform in the '*rbf*' kernel makes the already amplified expression values of two biological samples has zero or approximately zero distance in the feature space, i.e., the corresponding kernel matrices are identity or near identity matrices. In other words, the SVM learning machine inevitably loses its detection capabilities. Interestingly, it seems that the over-fitting problem can be overcome by setting a 'fine' threshold (e.g., τ=10) where only the least frequency signals are captured in MICA. For example, the MICA-SVM algorithm with the '*bior4.4'* wavelet' under the '*rbf*' kernel can achieve average classification rates 90.64% (sensitivity: 78.78%, specificity: 100%) for the colorectal data, 96.11% (sensitivity: 91.84%, specificity: 99.59) for the ovarian-qaqc data, and 94.38% (sensitivity: 90.62%, 98.88%) for the prostate data under the 100 trials of 50% holdout cross-validations. However, our simulations suggest that proteomic pattern classification is a linear separable problem and the '*linear*' kernel is an optimal kernel selection in the SVM-based algorithms.
