# Supplementary material for: A high performance profile-biomarker diagnosis for mass spectral profiles
Source: BMC Syst Biol. 2011 Dec 14;5(Suppl 2):S5. doi: 10.1186/1752-0509-5-S2-S5 (PMC3287485; doi:10.1186/1752-0509-5-S2-S5)
Supplement: Additional file 2 — Wavelet selection for MICA-SVM [file 1752-0509-5-S2-S5-S2.pdf]

Wavelet selections

Although only the wavelet '*db8*' is employed in our experiments, there is no other specific requirement for a wavelet except it should be orthogonal. To compare impacts of different wavelet selections on the algorithm performance, we select four family wavelets: 'd*b8*', '*sym8*', '*coif4*', and '*bior4.4*', in the classifications on the six profiles at the level threshold τ =3. Although it seems that there is no obvious classification advantage from one wavelet over the others under the 10-fold cross validation, the robust prior knowledge and less number of trials may have larger impact factors on the algorithm performance than a wavelet selection. However, we have found that the bi-orthogonal wavelet '*bior4.4*' and wavelet '*db8*' show some advantages over the others under the 100 trials of 50% holdout cross validations. Figure S2 illustrates our algorithm's average classification ratios and its standard deviations under the four wavelets. It seems that the wavelets *'db8'* and *'bior4.4'* show some advantages over the other two in achieving high-accuracy classifications.

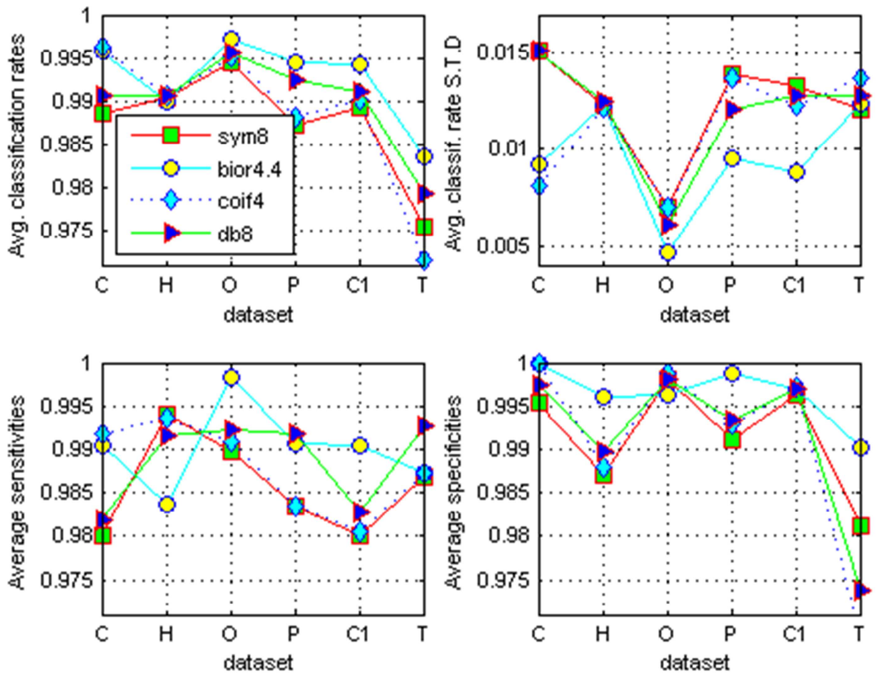

Figure S2 Comparisons on the average classification rates standard deviations of the average classification rates, average sensitivities and specificities for the MICA-SVM algorithm with four different wavelet selections under the 100 trials of 50% holdout cross validations on the six mass spectral profiles: ''C' (colorectal), 'H' (*hcc*) and 'O' (*ovarian-qaqc*), 'P' (*prostate*), and 'C1' ('*colorectal*'), 'T' ('*three-class*'). The wavelet *'db8'* and bi-orthogonal wavelet '*bior4.4'* show some advantages over the other.
